# A Genomic Background Based Method for Association Analysis in Related Individuals

**DOI:** 10.1371/journal.pone.0001274

**Published:** 2007-12-05

**Authors:** Najaf Amin, Cornelia M. van Duijn, Yurii S. Aulchenko

**Affiliations:** Department of Epidemiology and Biostatistics, Erasmus University Medical Center (MC) Rotterdam, Rotterdam, The Netherlands; Vrije Universiteit Medical Centre, Netherlands

## Abstract

**Background:**

Feasibility of genotyping of hundreds and thousands of single nucleotide polymorphisms (SNPs) in thousands of study subjects have triggered the need for fast, powerful, and reliable methods for genome-wide association analysis. Here we consider a situation when study participants are genetically related (e.g. due to systematic sampling of families or because a study was performed in a genetically isolated population). Of the available methods that account for relatedness, the Measured Genotype (MG) approach is considered the ‘gold standard’. However, MG is not efficient with respect to time taken for the analysis of genome-wide data. In this context we proposed a fast two-step method called Genome-wide Association using Mixed Model and Regression (GRAMMAR) for the analysis of pedigree-based quantitative traits. This method certainly overcomes the drawback of time limitation of the measured genotype (MG) approach, but pays in power. One of the major drawbacks of both MG and GRAMMAR, is that they crucially depend on the availability of complete and correct pedigree data, which is rarely available.

**Methodology:**

In this study we first explore type 1 error and relative power of MG, GRAMMAR, and Genomic Control (GC) approaches for genetic association analysis. Secondly, we propose an extension to GRAMMAR i.e. GRAMMAR-GC. Finally, we propose application of GRAMMAR-GC using the kinship matrix estimated through genomic marker data, instead of (possibly missing and/or incorrect) genealogy.

**Conclusion:**

Through simulations we show that MG approach maintains high power across a range of heritabilities and possible pedigree structures, and always outperforms other contemporary methods. We also show that the power of our proposed GRAMMAR-GC approaches to that of the ‘gold standard’ MG for all models and pedigrees studied. We show that this method is both feasible and powerful and has correct type 1 error in the context of genome-wide association analysis in related individuals.

## Introduction

Most of the complex genetic diseases have risk factors that are quantitative in nature. For instance, cholesterol level is a risk factor for myocardial infarction, Body mass index is a risk factor for type 2 diabetes. These quantitative traits (QTs) often have strong genetic determinants. It is therefore, of considerable interest to map the genes underlying QTs [1]. For most QTs relevant for human health and disease a large proportion–ranging from 30 to 80%–of variation is explained by genetic factors. Multiple genes are expected to contribute to this variation. The proportion of variation explained by a single gene is expected, however, to be small (less than 5%). For example, one of most known quantitative trait loci (QTLs), APOE is strongly and consistently associated with increased total cholesterol levels. Yet it explains only about 2–5% of the variation of this trait [2], [3]. When effects of specific common variants are expected to be small, association analysis provides a powerful approach to identify the gene compared to linkage analysis [4]. Genome-wide association analysis is a powerful tool to disentangle the complexity of quantitative traits, even in family based studies.

For pedigree-based association analysis several methods and software packages have been developed that utilize information about transmission of alleles, such as the orthogonal test for within-family variation [5] and family-based association test [6], [7]. As these methods analyze within-family variation, they are robust to population stratification. However, these methods ignore a large proportion of information provided by the between-family variation leaving room for improvement.

A conventional polygenic model of inheritance, which is a statistical genetics' “gold standard”, is a mixed model
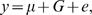
where *μ* is the overall mean, *G* is the vector of random polygenic effect, and *e* is the vector of random residuals. This model may be extended to study association by including a *kg* term, where *k* is the marker genotype effect, and *g* is the vector of genotypes. Such a model is known as the measured genotype (MG) model [8]. The MG approach, implemented using (restricted) maximum likelihood, is a powerful tool for the analysis of QTs when ethnic stratification can be ignored [9], [10] and pedigrees are small or when there are few dozens or hundreds of candidate polymorphisms to be tested. This approach, however, is not efficient in terms of computation time. This hampers the application of MG in genome-wide association analysis.

We proposed a fast two-step approximation to MG, a Genome Wide Rapid Association using Mixed Model and Regression (GRAMMAR) [11] . In the first step the individual environmental residuals are estimated, using additive polygenic model. Then the test of association is performed using these familial correlation-free residuals with a rapid least squares or score method. Though the two-step method is indeed computationally efficient and outperforms family based approaches like FBAT and QTDT in terms of power and speed, it loses power compared to MG [11]. The test becomes increasingly conservative and less powerful with the increase in the number of large full-sib families and increased heritability of the trait. Interestingly, empirical power of GRAMMAR is very close to that of MG.

Both classical MG and GRAMMAR approaches rely heavily on the availability, completeness, and correctness of genealogical information. When these assumptions are violated, the most likely outcome is inflation in type 1 error. Practically, genealogical information may often be available only for a limited number of generations, it may be inaccurate, and it may become increasingly inaccurate back in time. This may be taken as an argument for application of underpowered TDT-like methods to avoid false positive or negative results.

We and others reason that genomic data can be used to correct for the (only partly observed) true genealogy. With the new array technologies, large numbers of markers can be typed over the genome. These provide information on ‘genomic background’, which can be used to infer population (sub)structure and relations between study participants, which is classically done using the genealogy. In a recent study of type 2 diabetes, genomic control (GC) was applied to control for relatedness among cases and controls from Icelandic population [12], However, the type 1 and 2 errors of GC were not yet systematically investigated in the context of pedigree data analysis. In this study we aim to exploit the ideas of genomic background to extend our work on family based association [11] and determine how powerful and efficient the method for genome-wide association analysis of QTs in samples of related individuals is.

## Methods

Only minor proportion of markers in a genome-wide association study is expected to be truly associated with an analysis trait, and a vast majority of the markers may be thought of as realizations under the null hypothesis and can be used to characterize the null distribution of the test statistics. This idea follows that of Genomic Control (GC) method [13], which was introduced in the context of association analysis in population-based studies, where population stratification and cryptic relatedness may be present.

Following Devlin and Roeder [13], [14] we suggest estimation of the test statistic inflation factor λ by regressing the trait on *N* loci. From each regression analysis, estimate *T_i_^2^ = β_i_^2^/Var(β_i_)*, where *β_i_* is the effect of the *i-*th SNP (*i* from 1 to *N*) and *Var(β_i_)* is the variance of the estimate. Inflation factor is estimated as
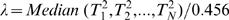
where 0.456 is the median of the *χ_1_^2^* distribution with a non-central variance equal to *φ*. The number of loci used, *N*, in a genome-wide association study is typically reflecting all loci investigated or the ones generating lowest 95% of test statistics [15], [16]. It should be noted that the value of λ in conventional GC is constrained to be greater than one as values less than one have no theoretical meaning.

We propose use of GC to correct for conservativeness of the GRAMMAR approach outlined earlier. This method which we call GRAMMAR-GC involves three steps: (a) trait heritability is estimated by using trait and pedigree data using the following mixed model
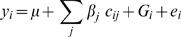
where *β_j_* is the effect of *j* ^th^ covariate , c*_ij_* is the value of *j^th^* covariate and *μ*, *G*, and *e* are defined earlier. And environmental residuals are estimated as
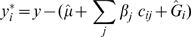
(b) the markers are tested for association with these residuals using simple linear regression
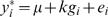
where *k* and *g* are defined earlier

(c) GC procedure is applied to correct the test statistic. The deflation factor ζ is estimated by regressing residuals from step (a) on each of the *k* null loci and from each regression analysis 
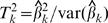
 is estimated, where 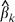 is the effect of the *k^th^* SNP. The deflation factor ζ is estimated as 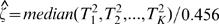. Then *T^2^/* 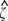 is compared with *χ* ^2^_(1)_ to determine whether the locus is significantly associated with the QT [14].

Steps (a) and (b) comprise the original GRAMMAR approach, leading to a conservative test. In step (c), GC is used to estimate the deflation factor ζ. This deflation factor is estimated in exactly the same way Bacanu et al. [14] estimate inflation factor λ for quantitative traits. The only difference is that ζ<1 in contrast to λ in conventional GC methods which is constrained to be >1. This difference comes from the fact that residuals from step (a) are regressed on null loci to obtain the estimate of ζ instead of original trait data as in conventional GC.

The original GRAMMAR relies on the availability of a precise and complete pedigree structure for heritablity estimation in step (a). This can, however, be done by using kinship coefficients estimated from genomic data where the genomic estimate of kinship for a pair of individuals *i* and *j* is obtained using the formula [17]

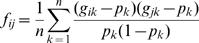
where *g_ik_* is the genotype of the *i*-th person at the *k*-th SNP of the (coded as 0, 1/2 and 1, for rare allele homozygote, heterozygote and common homozygote, respectively), *p_k_* is the frequency of the major allele, and *n* is the number of SNPs used for kinship estimation.

Heritability is then estimated by maximizing the log-likelihood of the data
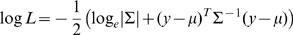
where *y* is the vector of trait values, *μ* is the mean and *Σ = Φσ_G_^2^+Iσ_e_^2^* is the variance-covariance matrix. Here, *Φ* is the relationship matrix whose elements are defined as 2 *f_ij_*, *σ_G_^2^* is the additive genetic variance due to polygenes, *I* is the identity matrix and *σ_e_^2^* is the residual variance. Trait environmental residuals were obtained as
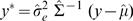



To avoid confusion we refer to the new method as Pedigree GRAMMAR-GC (PedGR-GC) when in step (a) heritability is estimated from the genealogy, and Genomic GRAMMAR-GC (GenGR-GC) when the heritability is estimated from the genomic data. For PedGR-GC, environmental residuals were estimated using ASReml [18]. All other computations were performed using freely available R software (http://www.r-project.org); computations associated with GenGR-GC were facilitated by GenABEL package for R [19], implementing procedures to compute genomic kinship, maximize polygenic models and compute the residuals.

### Pedigrees used

To investigate type 1 error and power of the proposed methods we used three different pedigree structures representing three different study scenarios. For example, Nuclear pedigrees (NP) simulated a study performed in the outbred population, the Erasmus Rucphen Family study (ERF) population is a study of a genetically isolated population and Idealized Pig Population (IPP) simulates a livestock population.

**NP:** 337 sib-trios each having 3 phenotyped and genotyped siblings; in total, 1011 individuals were available for the analysis. The founders in each pedigree were assumed to be unrelated.

**ERF:** 1010 phenotyped and genotyped individuals all related to each other in a single large complex pedigree of about 10,000 individuals. The pedigree extends up to 23 generations and contains thousands of loops. The phenotyped individuals are a part of Erasmus Rucphen Family (ERF) study, performed in a young genetically isolated Dutch population [20].

**IPP:** idealized pig population, consisting of 10 sires, each mated with 10 dams, nine of which have 10 and one 11 measured offspring. Thus each sire has 101 half-sib offspring in families of 10 full-sibs. In total 1010 phenoytped individuals were available for the analysis.

Genetic data was simulated using each of these pedigrees under several models. The SNP that was analyzed for association had a minor allele frequency of 10%. For studying type 1 error this SNP was not associated with the trait while for studying power this SNP explained 1, 2, or 3% of the total trait variation and acted in an additive manner. The total heritability of the trait was set to 30, 50, and 80% and this included the variation due to the QTL studied. To enable genomic control, we also simulated 200 unlinked SNPs for each realization.

These pedigrees and models were used to evaluate original GRAMMAR and thus we can directly compare type 1 error and power of the suggested methods to these evaluated by Aulchenko et al [11], namely, MG, DFS (linear regression which does not take family structure into account), QTDT and FBAT.

## Results

Table 1 shows the 95^th^ percentile of the test statistic and type 1 error (proportion of simulations that resulted in a χ^2^≥3.84 and χ^2^≥6.64, corresponding to tabulated α = 0.05 and 0.01 respectively) for GC and PedGR-GC and GRAMMAR. For GC and PedGR-GC type 1 error is close to the nominal α while GRAMMAR is conservative and this conservativeness increases with the increase in the number of sibships and the heritability of the trait.

**Table 1:** 95^th^ percentiles of the distribution and type 1 error for PedGR-GC, GC and GRAMMAR.

						Type 1 error at a given threshold							
Pedigree			95^th^ percentile			χ^2^≥3.84			χ^2^≥6.64				
	q_snp_	h^2^	PedGR-GC	GC	GRAMMAR	PedGR-GC	GC	GRAMMAR	PedGR-GC	GC	GRAM MAR	λ±SE(λ)	ζ±SE(ζ)
												GC	PedGR-GC
NP													
	0.1	0.3	3.97	3.90	3.25	0.053	0.051	0.029	0.014	0.013	0.010	1.16±0.006	0.88±0.004
	0.1	0.3q	3.89	3.71	3.11	0.050	0.045	0.034	0.012	0.010	0.004	1.16±0.006	0.88±0.005
	0.1	0.5	3.61	3.70	2.89	0.040	0.048	0.021	0.004	0.006	0.002	1.26±0.007	0.82±0.004
	0.1	0.8	3.85	4.17	2.92	0.050	0.056	0.026	0.017	0.016	0.006	1.41±0.007	0.72±0.004
	0.5	0.3	3.92	3.87	3.35	0.050	0.052	0.039	0.010	0.014	0.003	1.17±0.006	0.88±0.005
	0.5	0.3q	3.93	3.73	3.14	0.051	0.048	0.033	0.011	0.009	0.005	1.15±0.006	0.88±0.005
	0.5	0.5	3.65	4.01	2.80	0.045	0.056	0.027	0.008	0.009	0.003	1.26±0.007	0.81±0.004
	0.5	0.8	3.89	4.12	2.7	0.052	0.059	0.024	0.011	0.008	0.003	1.42±0.007	0.72±0.004
ERF													
	0.1	0.3	3.85	3.78	3.08	0.050	0.047	0.031	0.009	0.010	0.004	1.29±0.008	0.86±0.005
	0.1	0.3q	3.93	3.83	3.09	0.053	0.049	0.034	0.006	0.004	0.002	1.27±0.008	0.85±0.005
	0.1	0.5	3.93	3.86	3.15	0.055	0.050	0.023	0.010	0.009	0.003	1.47±0.009	0.79±0.004
	0.1	0.8	3.95	3.90	2.71	0.052	0.051	0.021	0.012	0.009	0.001	1.71±0.010	0.69±0.003
IPP													
	0.1	0.3	4.08	3.83	2.73	0.056	0.049	0.019	0.017	0.008	0.003	3.26±0.037	0.68±0.003
	0.1	0.3q	3.66	3.06	2.43	0.043	0.026	0.014	0.011	0.007	0.001	3.19±0.046	0.68±0.004
	0.1	0.5	4.14	3.70	2.58	0.056	0.046	0.012	0.011	0.012	0.004	4.72±0.053	0.63±0.003
	0.1	0.8	4.13	3.75	2.27	0.059	0.045	0.012	0.014	0.009	0.000	7.03±0.077	0.58±0.003

Pedigree studied NP: 337 nuclear families; ERF:1010 in one large pedigree; IPP: idealized pig population

h^2^: total heritability; total heritability of 0.3q represents heritability of 0.3 explained by a single unlinked QTL

q_snp_: minor allele frequency of the SNPs studied

λ: estimate of the inflation factor for genomic control

ζ: estimate of the deflation factor for GRAMMAR-GC

**Figure 1:**
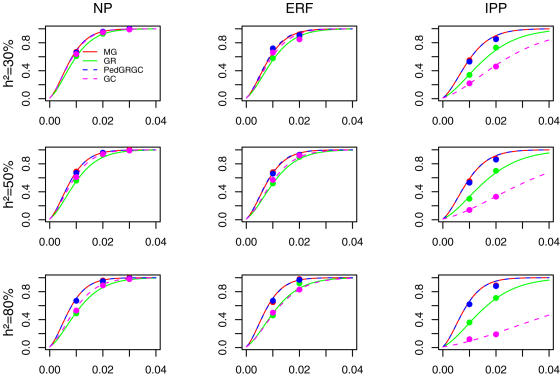
Power of MG (red line), GRAMMAR (green line), PedGR-GC (blue dashed line), and GC (pink dashed line) to detect association under different heritability models and pedigree structures. The three rows show the power under different heritability models (from 30% to 80%) and the three columns show power achieved in different pedigrees namely nuclear pedigrees (NP), Erasmus Rucphen Family (ERF), and idealized pig population (IPP). The y-axis of each panel shows power while the x-axis shows the proportion of variance explained by the QTL under study. The red (for MG), green (for GRAMMAR), blue (for PedGR-GC), and pink (for GC) circles show the empirical power estimates. The power estimates are based on α = 0.01. The empirical power estimates are based on 1000 simulations for NP, and IPP, and 100 simulations for ERF.

Supplementary Table S1 and Figure 1 illustrate the power of the proposed (GC and PedGR-GC) and previous methods (MG and GRAMMAR). In the Figure 1, power to reach χ^2^≥6.64 (α = 0.01) is depicted with circles. From the available evaluation points we also estimated the slope of linear regression of non-centrality parameter on proportion of variance explained and used this slope to derive power curves.

Figure 1 shows that the power of PedGR-GC (blue dashed line) is very close to the power of the ‘gold standard’ measured genotype approach (red line) for all scenarios. These two methods appear to be the most powerful of all methods for all pedigree structures and genetic models evaluated. The power of GC (pink dashed line) is close to that of MG and PedGR-GC when the heritability is low but its' power declines when the heritability of the trait increases or when pedigrees with large number of full-sib families (IPP) are investigated. GRAMMAR (green line) performs similar to GC in a sample of nuclear families and in the ERF sample, but is more powerful when IPP pedigree is investigated.

To study the potential of described methods on genome-wide scale we used 695 people who are a part of the ERF pedigree and who were genotyped using Illumina 6K SNP linkage panel. Based on pedigree records, the 695 people formed 471 pairs of first-, 311 pairs of second-, 681 pairs of third- and 1,105 pairs of forth-degree relatives and 223,578 pairs of more distant relationship.

We generated 500 replicas for each of the models assuming total trait heritability of 30, 50 and 80%. In each replica, we selected five hundred random SNPs each explaining equal proportion of variance amounting to the total heritability minus 4%, and one random SNP explaining 4% of the phenotypic variance. The analysis trait was obtained as a sum of the SNP effects and a random number from the Normal distribution with mean zero and variance 0.7, 0.5 or 0.2 for total trait heritabilities of 30, 50 and 80%, respectively.

Type one error was estimated as the proportion of non-associated SNPs (>2.5 million tests in total) showing association P-value of 5% or less. The statistical power was estimated as the proportion of replicas in which the SNP explaining 4% of variance passed genome-wide significant threshold (Bonferroni-corrected P-value 0.05/5524 = 9×10^−6^).

For analysis, we used GC, PedGR-GC and GenGR-GC. For GenGR-GC, the kinship matrix used was estimated from genomic information on 5524 autosomal SNPs typed in 695 members of the ERF study.

**Figure 2:**
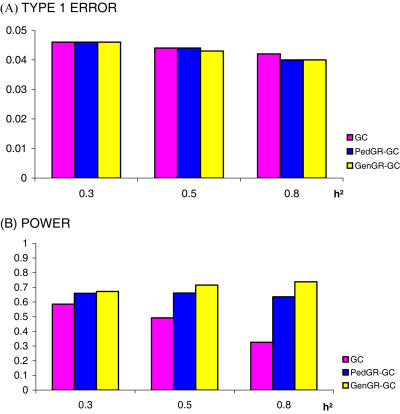
Type 1 error (A) and power (B) to achieve 5% genome-wide significance at the truly associated SNP in a study of 695 ERF people genotyped for 5524 autosomal SNPs.

All methods showed a genome-wide type 1 error which was very close, but lower than the pre-specified threshold of 5% (Figure 2A). The methods tended to be more conservative at higher heritabilities. These results are consistent with the observations of others, that GC is conservative, and can be explained by the fact that all SNPs, some of which were associated with the trait, are used to estimate the null distribution of the test statistic.

## Discussion

In this work we aimed to develop fast and powerful methods for genome-wide association analysis in samples of related individuals by exploiting the ideas of genomic background for correction of the distribution of the test statistics and for inferring the relation between study subjects. We show that methods, which exploit only genomic background, such as Genomic Control (GC) and GRAMMAR-GC using genomic kinship, are powerful and genome-wide feasible methods. Moreover, genomic GRAMMAR-GC, which infers genetic relations from genomic data, may be superior to the methods that use pedigree information in an exact manner.

Our simulations show that GC is a valid method to study data coming from samples of related individuals. GC can be a powerful tool for the analysis of pedigree based quantitative trait loci. It outperforms traditional family based approaches like QTDT and FBAT (cf. Table 1 & Table 2 of Aulchenko et. al.[11]). The power of GC is close to that of the ‘gold standard’ measured genotype approach when trait heritability is low or moderate and human pedigrees are studied. However, the power of GC drops notably with high trait heritability and when pedigrees involve very large sibships, which are likely to be observed in animal pedigrees.

The results that GC was less powerful, than GRAMMAR-GC based methods, and tended to lose power at higher heritabilites (Figure 2B), are not surprising and are consistent with our previous findings [11]. Interestingly enough in a study of real Genome-wide data in ERF pedigree, GenGR-GC was consistently more powerful than pedigree-based GRAMMAR-GC (PedGR-GC), and the power advantage became more pronounced at higher heritabilities.

We proposed an extension to the GRAMMAR method [11], which increases its' power, maintains a nominal type 1 error and also does not require the precise knowledge of pedigree structure. Our method (GRAMMAR-GC) involves three steps which include removing the correlation from the data using relationship matrix estimated from either the pedigree or the genome (derivation of environmental residuals), using the correlation-free residuals from step 1 as the trait to perform association analysis, and then applying GC to correct the test statistic. We show through simulations that our proposed method performs very similar to the Measured Genotype (MG) approach with respect to type 1 error and power yet it is fast and feasible for genome-wide association analysis. By analysis of real genome-wide SNP data we showed that when the genomic data is used to estimate the relationship matrix (GenGR-GC) instead of the estimate obtained from genealogy, the power might be even improved.

One of our conclusions is that in genome-wide association studies of related individuals genomic background based methods such as genomic GRAMMAR-GC should be preferred over the ones exploiting known pedigree structure, such as pedigree GRAMMAR-GC or MG approach. There are two reasons why we believe that GenGR-GC should be preferred over its' pedigree analog.

First, errors in genealogy such as mis-specification of relations can lead to either false positives or negatives. Secondly, relationship coefficient computed from a pedigree is an expectation of the proportion of genome shared identical by descent (IBD) under the infinitesimal model, assuming infinite number of unlinked loci. The true proportion of genome shared, however, may deviate from this expectation [17]. For example, for remote relatives there is a fair chance of not sharing any genomic loci IBD. We may speculate that kinship estimated based on marker data can reflect the true unobserved genomic sharing better then the expectation computed from even a correct pedigree. If this is true, under the polygenic model we should expect that methods based on kinship estimated from marker data will be more powerful than the methods estimating kinship from the pedigree. We however leave a more detailed investigation of effects of pedigree error and precision of genomic kinship estimates on type 1 error and power to future works.

Another advantage of the GRAMMAR-GC is that the environmental residuals used for the analysis are free from familial correlations. Therefore the structure of the data becomes exchangeable. This means that permutation techniques may be applied to derive empirical measures of significance. In the analysis of data where adjacent SNPs are correlated due to linkage disequilibrium, thresholds set via permutation will account for these correlations and result in less stringent thresholds than those set by Bonferroni correction. This will become more and more important in the future, when denser marker maps with millions of SNPs will be applied to do association studies. Another attractive feature is that a range of new methods developed for classical “unrelated individuals” design can be applied to polygenic residuals obtained at the first stage of the analysis. In recent years, much progress was made in development of powerful methods and software which are robust to possible allelic heterogeneity through utilization of haplotype clustering and population genetic coalescence modeling [21], [22].

Finally, GRAMMAR-GC is easily extendable: for example, it is easy to include covariates, interactions with sex and environment, gene-gene interactions and parent of origin effects.

To conclude, GRAMMAR-GC is a fast powerful approach for genome-wide association analysis of quantitative traits in samples of related individuals, which does not require precise knowledge of pedigree structure. This method is implemented as part of the GenABEL package, available at http://cran.r-project.org/.

## Supporting Information

Table S1 Mean χ2 statistics and proportion of the test statistics ≥tabular critical value.
(0.05 MB DOC)
