# Supplementary material for: A Genomic Background Based Method for Association Analysis in Related Individuals
Source: PLoS One. 2007 Dec 5;2(12):e1274. doi: 10.1371/journal.pone.0001274 (PMC2093991; doi:10.1371/journal.pone.0001274)
Supplement: Table S1 — Mean χ2 statistics and proportion of the test statistics ≥tabular critical value. (0.05 MB DOC) [file pone.0001274.s001.doc]

**Supplementary table 1:** **Mean** **χ^2^ statistics and proportion of the test statistics ≥ tabular** **critical value**

|  | | | | Proportions of simulations | | | |  | |
| --- | --- | --- | --- | --- | --- | --- | --- | --- | --- |
| PED | | M[Χ^2^] | | χ^2^≥3.84 | | χ^2^≥6.64 | |  | |
| QTL | h^2^ | PedGR-GC | GC | PedGR-GC | GC | PedGR-GC | GC | λ ± SE(λ)  GC | ζ ± SE(ζ)  PedGR-GC |
| NP |  |  |  |  |  |  |  |  |  |
| 0.01 | 0.3 | 10.35 | 10.0 | 0.83 | 0.82 | 0.66 | 0.64 | 1.16±0.007 | 0.88±0.005 |
|  | 0.5 | 10.21 | 9.32 | 0.85 | 0.82 | 0.67 | 0.61 | 1.26±0.007 | 0.81±0.004 |
|  | 0.8 | 10.66 | 8.32 | 0.87 | 0.76 | 0.67 | 0.53 | 1.43±0.008 | 0.73±0.004 |
| 0.02 | 0.3 | 19.90 | 19.2 | 0.99 | 0.99 | 0.96 | 0.95 | 1.17±0.006 | 0.88±0.005 |
|  | 0.5 | 19.60 | 18.1 | 0.99 | 0.98 | 0.96 | 0.94 | 1.26±0.007 | 0.82±0.004 |
|  | 0.8 | 20.38 | 16.3 | 0.99 | 0.97 | 0.95 | 0.89 | 1.41±0.007 | 0.72±0.004 |
| 0.03 | 0.3 | 29.57 | 29 | 1 | 1 | 1 | 0.99 | 1.15±0.006 | 0.88±0.005 |
|  | 0.5 | 29.45 | 26.7 | 1 | 1 | 0.99 | 0.99 | 1.27±0.007 | 0.81±0.004 |
|  | 0.8 | 30.0 | 23.8 | 1 | 1 | 0.99 | 0.98 | 1.42±0.007 | 0.73±0.004 |
| ERF |  |  |  |  |  |  |  |  |  |
| 0.01 | 0.3 | 10.10 | 9.24 | 0.84 | 0.84 | 0.72 | 0.66 | 1.25±0.025 | 0.83±0.014 |
|  | 0.5 | 10.23 | 8.21 | 0.81 | 0.70 | 0.66 | 0.57 | 1.48±0.027 | 0.78±0.013 |
|  | 0.8 | 10.10 | 7.28 | 0.83 | 0.72 | 0.67 | 0.50 | 1.77±0.033 | 0.72±0.010 |
| 0.02 | 0.3 | 19.20 | 17.3 | 0.97 | 0.95 | 0.91 | 0.85 | 1.24±0.023 | 0.84±0.016 |
|  | 0.5 | 19.48 | 15.8 | 0.99 | 0.99 | 0.93 | 0.92 | 1.47±0.025 | 0.80±0.015 |
|  | 0.8 | 20.90 | 13.5 | 0.99 | 0.95 | 0.97 | 0.83 | 1.75±0.031 | 0.71±0.010 |
| IPP |  |  |  |  |  |  |  |  |  |
| 0.01 | 0.3 | 8.41 | 4.19 | 0.75 | 0.42 | 0.53 | 0.22 | 3.3±0.035 | 0.69±0.004 |
|  | 0.5 | 8.55 | 3.20 | 0.76 | 0.29 | 0.53 | 0.14 | 4.78±0.052 | 0.64±0.003 |
|  | 0.8 | 10.30 | 2.79 | 0.80 | 0.26 | 0.62 | 0.12 | 6.86±0.075 | 0.58±0.003 |
| 0.02 | 0.3 | 15.96 | 7.35 | 0.95 | 0.70 | 0.85 | 0.46 | 3.29±0.036 | 0.68±0.004 |
|  | 0.5 | 17.00 | 5.72 | 0.94 | 0.55 | 0.86 | 0.33 | 4.71±0.051 | 0.63±0.003 |
|  | 0.8 | 18.56 | 3.87 | 0.95 | 0.40 | 0.88 | 0.19 | 7.00±0.075 | 0.59±0.003 |

QTL: proportion of trait variation explained by the locus under study

h^2^: total heritability

λ: estimate of the inflation factor for genomic control

ζ: estimate of the deflation factor for GRAMMAR-GC
